# Solvent: A Key in Digestive Ripening for Monodisperse Au Nanoparticles

**DOI:** 10.1186/s11671-016-1797-7

**Published:** 2017-01-09

**Authors:** Peng Wang, Xuan Qi, Xuemin Zhang, Tieqiang Wang, Yunong Li, Kai Zhang, Shuang Zhao, Jun Zhou, Yu Fu

**Affiliations:** 1 College of Sciences, Northeastern University, Shenyang, 110004 China; 2 School of Materials Science and Engineering, Key Laboratory for Anisotropy and Texture of Materials, Ministry of Education, Northeastern University, Shenyang, 110004 China

**Keywords:** Solvent polarity, Digestive ripening, Au nanoparticles, Monodispersity

## Abstract

This work has mainly investigated the influence of the solvent on the nanoparticles distribution in digestive ripening. The experiments suggested that the solvents played a key role in digestive ripening of Au nanoparticles (Au NPs). For the benzol solvents, the resulting size distribution of Au NPs was inversely related to the solvent polarity. It may be interpreted by the low Gibbs free energy of nanoparticles in the high polarity medium, which was supposedly in favor of reducing the nanoparticles distribution. Through digestive ripening in the highly polar benzol solvent of *p*-chlorotoluene, monodisperse Au NPs with relative standard deviation (RSD) of 4.8% were achieved. This indicated that digestive ripening was an effective and practical way to prepare high-quality nanoparticles, which holds great promise for the nanoscience and nanotechnology.

**Electronic supplementary material:**

The online version of this article (doi:10.1186/s11671-016-1797-7) contains supplementary material, which is available to authorized users.

**Graphical Abstract:**
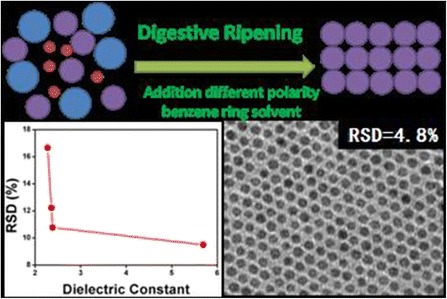
The polarity of benzol solvent plays significant role in obtaining high-quality monodisperse Au nanoparticles.

## Background

Au nanoparticles (Au NPs) and their self-assemblies have drawn intense attentions due to their unique properties and potential applications [1, 2], such as catalysts [3], electronic and optoelectronic nanodevices [4], biosensors [5], and biomedicine [6]. For fully implementing the functions of Au NPs, the uniform size distribution is of key significance. The monodisperse nanoparticles (relative standard deviation less than 5.0%) show unique properties and higher performances compared with the corresponding polydisperse ones [7, 8]. Unfortunately, it is hard to prepare monodisperse Au NPs with simple synthesis process and common starting materials [9–11]. Therefore, a great deal of effort has been made to obtain monodisperse Au NPs [12]. One main strategy is direct alteration of the synthesis method, including selection of special metal sources and reduction agents, introduction of nanoparticle seeds, addition of surfactants, and so on. However, although the monodisperse Au NPs have been achieved by direct synthesis [13], it suffers from high-cost of starting materials, tedious separation and unsatisfied repeatability. An alternative approach to preparing monodisperse Au NPs is post-treatment of the prepared ones, which could detour the issue.

Digestive ripening, discovered by Lin [14], is an effective post-treatment method. It is carried on by refluxing a nanoparticle suspension with an excess amount of capping agents (called as digestive ripening agents (DRA)), which could cause the shrink of large particles and the growth of small particles to achieve an equilibrium size at a stable state [11, 12]. A distinct feature of digestive ripening is that it can obtain high reproducibility and yield with fine control [15]. The effect factors on digestive ripening, including capping agent, temperature, refluxing time, field effect, and the length of digestive ripening agent, have been explored [11, 16–20]. Solvent is an important participant in digestive ripening. Moreover, the study has shown the solvent plays a vital role in the synthesis process, which implied the effect of the solvent on digestive ripening would be remarkable. However, the related work has been rarely reported. In addition, thus far the monodisperse Au nanoparticles obtained by digestive ripening mainly came from the system reported by Lin [11]. In that system, the original Au nanoparticles were synthesized by a surfactant-assisted method. Its process was tedious and the yield was low, that substantially weakened the advantage of digestive ripening.

Based on the above consideration, digestive ripening of Au NPsin different solvents was investigated in this work. The experiments showed that there was no obviously development in size distribution of Au NPs in the solvents of linear hydrocarbon. However, there was a dramatic change when benzol solvents were used. Moreover, the distribution was closely related with the solvent polarity. The higher the polarity of the used solvent is, the lower the RSD of the resulting nanoparticles is. When a highly polar benzol of *p*-chlorotoluene was used as the reflux solvent, RSD of the Au NPs after digestive ripening could reach as low as 4.8%, with which the superlattices could be assembled. Additionally, it is worth to emphasize that the original preparation of the Au NPs in our study was fast, low-costed, and surfactant-free. Combined with the simple synthesis method, the work offered a promising approach to facile fabrication of the monodisperse Au nanoparticles.

## Methods

### Synthesis of Initial Au NPs

The DDT-capped gold NPs was synthesized according to the literature [21]. Firstly, 50 mM AuCl_4_^−^/H^+^ solution was prepared by dissolving HAuCl_4_·4H_2_O with the same molar amount of HCl in aqueous. 50 mM BH_4_^−^/OH^−^ solution was made by dissolving NaBH_4_ with the same molar amount of NaOH in aqueous. Secondly, 475 μL of AuCl_4_^−^/H^+^ solution was diluted with 47 mL DI water. Then, BH_4_^−^/OH^−^ (2.125 mL of 50 mM) was added to the solution under magnetically stirring, followed by heating for 3–4 min at boiling temperature of water. After it was cooled to room temperature, acetone (32 mL), hexane (38 mL), and DDT (0.5 μL) was introduced into the solution and mixed vigorously by hands for about 1 min to transfer Au NPs to the organic phase. Finally, the mixed solution was separated by a separating funnel, and the initial Au NPs were obtained.

### Digestive Ripening of Au NPs

In a typical digestive ripening, a batch of as-prepared Au NPs solution was evaporated for about 35 °C by rotary evaporation to get rid of the solvent. And then, the dried Au NPs were re-dispersed in 10 mL different solvents (*n*-hexane, toluene, *m*-xylene, *p*-xylene, chlorobenzene, *p*-chlorotoluene and *n*-octane) with the addition of 2.5 μL DDT, respectively. Those colloids were refluxed at boiling point of different solvents and then slowly cooled down. The whole process of digestive ripening was completed.

## Results and Discussion

**Fig. 1:**
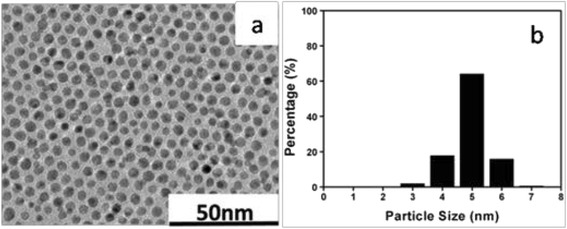
**a** TEM image of the initial Au nanoparticles. **b** The corresponding particle size histogram

In this study, we chose a two-step method to synthesize DDT-coated Au NPs. The first step was preparing naked Au NPs in an aqueous solution by reduction of HAuCl_4_ by NaBH_4_. The second was coating the NPs with DDT by extracting the naked NPs into a DDT solution in hexane. Thanks to the low-costed starting materials and facile process, this method was an effective way to prepare at a large scale. Unfortunately, the polydispersity of the resulting nanoparticles was still unsatisfied. As shown in Fig. 1, the average diameter of the Au NPs was 4.47 nm and the RSD was 15%.

**Fig. 2:**
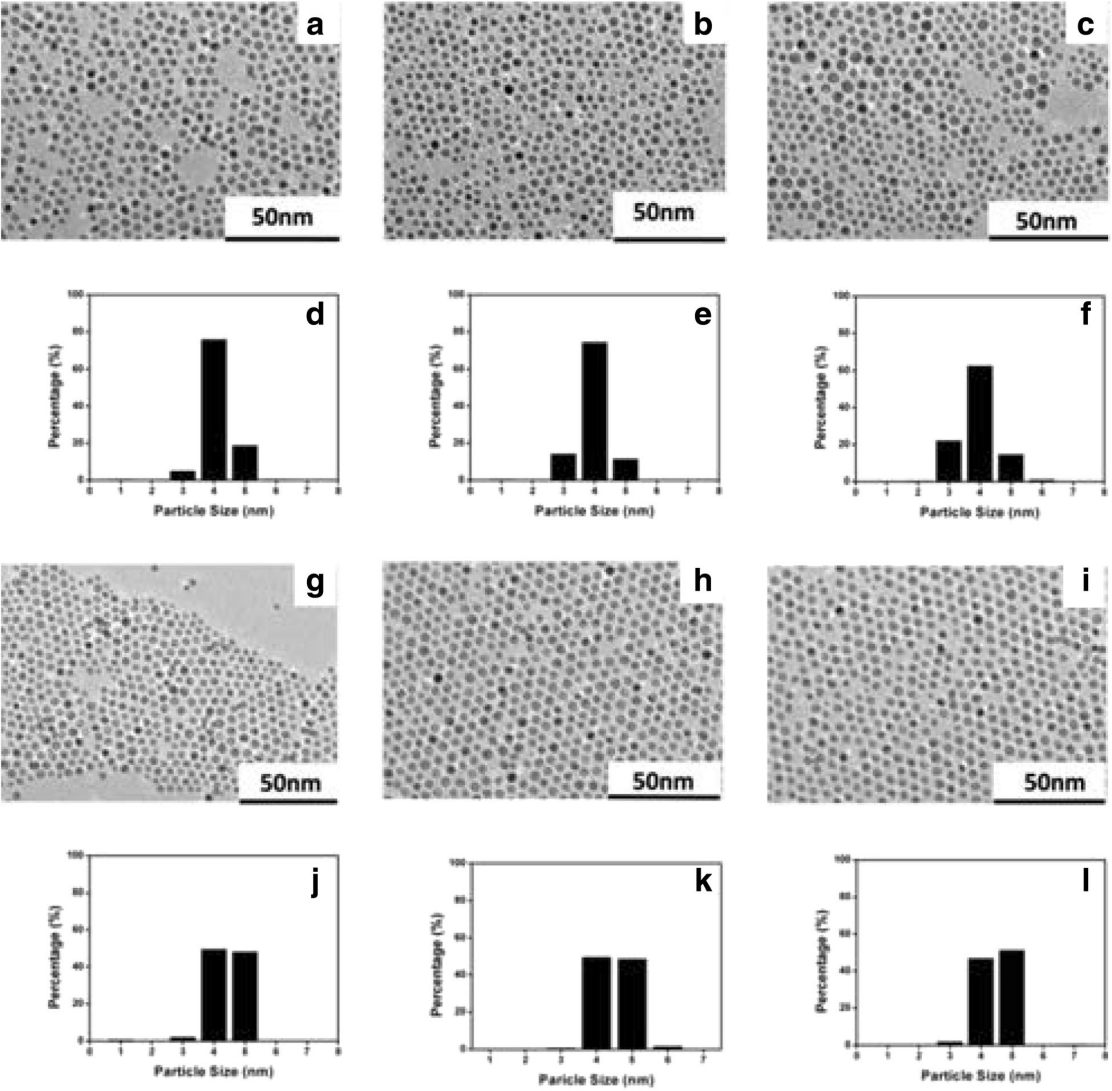
TEM images of the Au NPs after 4 h digestive ripening in different solvents. **a** *n*-Hexane. **b** *n*-Octane. **c** *p*-Xylene. **g** *m*-Xylene. **h** Toluene. **i** Chlorobenzene. The corresponding particle size histograms. **d** *n*-Hexane. **e** *n*-Octane. **f** *p*-Xylene. **j** *m*-Xylene. **k** Toluene. **l** Chlorobenzene

Because NaBH_4_was used as the reduction agent, the synthesis process of the Au nanoparticles was fast and almost uncontrollable; it would be difficult to further improve the distribution by optimizing synthesis conditions. Therefore, post-treatment of the resulting nanoparticles was an alternative strategy to improve the size distribution. In this work, we employed the digestive ripening as the post-treatment and mainly explored the influence of the solvents. Digestive ripening was carried on by reflux of the prepared Au NPs in different solvents (*n*-hexane, toluene, *p*-xylene, *m*-xylene, chlorobenzene, and *n*-octane) at their respective boiling temperatures. The TEM images and corresponding distribution analysis of the Au NPs after 4 h digestive ripening were shown in Fig. 2, and the related data were summarized in Table 1.

**Table 1:** A summary of Au NPs reflux in different solvents

Solvent	Particle size (nm)	RSD (%)	Temperature of boiling point (°C)	Dielectric constant (20 °C)^25^
*n*-Hexane	3.66 ± 0.45	12	68	1.890
*n*-Octane	3.47 ± 0.48	14	125	1.948
*p*-Xylene	3.42 ± 0.57	17	138	2.273
*m*-Xylene	3.92 ± 0.48	12	139	2.359
Toluene	3.99 ± 0.43	11	110	2.385
Chlorobenzene	4.00 ± 0.38	9.5	136	5.690

In digestive ripening of the Au NPs, there were two possible factors that could play the vital role. The first possible one was the digestive ripening temperature, i.e., the reflux temperature of the solvent. However, as shown in Table 1, there was not a direct relationship between the reflux temperature and the size distribution. For example, the reflux temperatures of *p-*xylene, *m*-xylene, and chlorobenzene were almost the same, but the size distribution of the Au NPs after digestive ripening in them was completely different, ranging from 9.5 to 17%. For the other solvents, including *n*-hexane, toluene, and *n*-octane, the distribution also varied with the reflux temperatures at random suggesting that the digestive ripening temperature was not directly related with the size distribution.

The second possible factor could be the physical property of the solvent. The solvent we used in digestive ripening can be classified into two categories: linear solvents and benzol solvents. As shown in Table 1, the linear solvents (*n*-hexane, *n*-octane) could not remarkably improve the nanoparticles distribution. This was likely because the flexible chain of linear solvents could intertwine with the flexible chain of DDT coated on the nanopartcles, which reduced the etching degree of the Au NPs in digestive ripening. As a result, the monodisperse Au NPs was hard to obtain. On the contrary, the size distribution may dramatically be improved after digestive ripening in benzol solvents (*p*-xylene, *m*-xylene, toluene, and chlorobenzene). RSD could decrease from the initial 15 to 9.5% after 4 h reflux in chlorobenzene. Moreover, it was found that the size distribution was inversely related to the polarity of solvents. As shown in Table 1, with the increase of dielectric constants (representing the solvent polarity [22]) of the solvents, the size distribution of the Au NPs after digestive ripening was decreased gradually.

The influence of the solvents polarity on the size distribution could be explained by the following arguments. The polydispersity of the nanoparticles is supposed to stem from the random inhomogeneity of the reflux system, which was caused by the uncontrolled environmental disturbance. So, the nanoparticles polydispersity should be determined by the relative environmental disturbance to the nanoparticles energy. Low relative disturbance means that the influence of the environmental disturbance to the nanoparticles is weak, leading to narrow distribution. In our case, for digestive ripening in different solvents, the intensity of the disturbance should be in the same level. Therefore, the energy of the nanoparticles in different solvents is the key factor to the polydispersity. The lower the nanoparticles energy is, the lower the relative environmental disturbance is. That indicates weak influence of the environments and low polydispersity.

Based on this consideration, the Gibbs free energy of a spherically charged particle was expressed as in Eq. 1 [23].

1 $$ G(r)=4\uppi \upsigma {r}^2+\frac{k{z}^2{q}^2}{2r} $$

2 $$ k=\frac{1}{4\uppi \varepsilon } $$

where σ is the interface free energy of the particle, *z* is the number of charges, *r* is the particle radius, and *k* is a constant related to the dielectric constants (*ε*) of the medium, as expressed in Eq. 2. In our study, the nanoparticles’ radius was almost the same. Therefore, the dielectric constant of the solvent was the main factor. Because of the inverse proportion of the dielectric constant to the free energy, the Au NPs in the solvent with higher polarity were more stable and could have lower polydispersity.

**Fig. 3:**
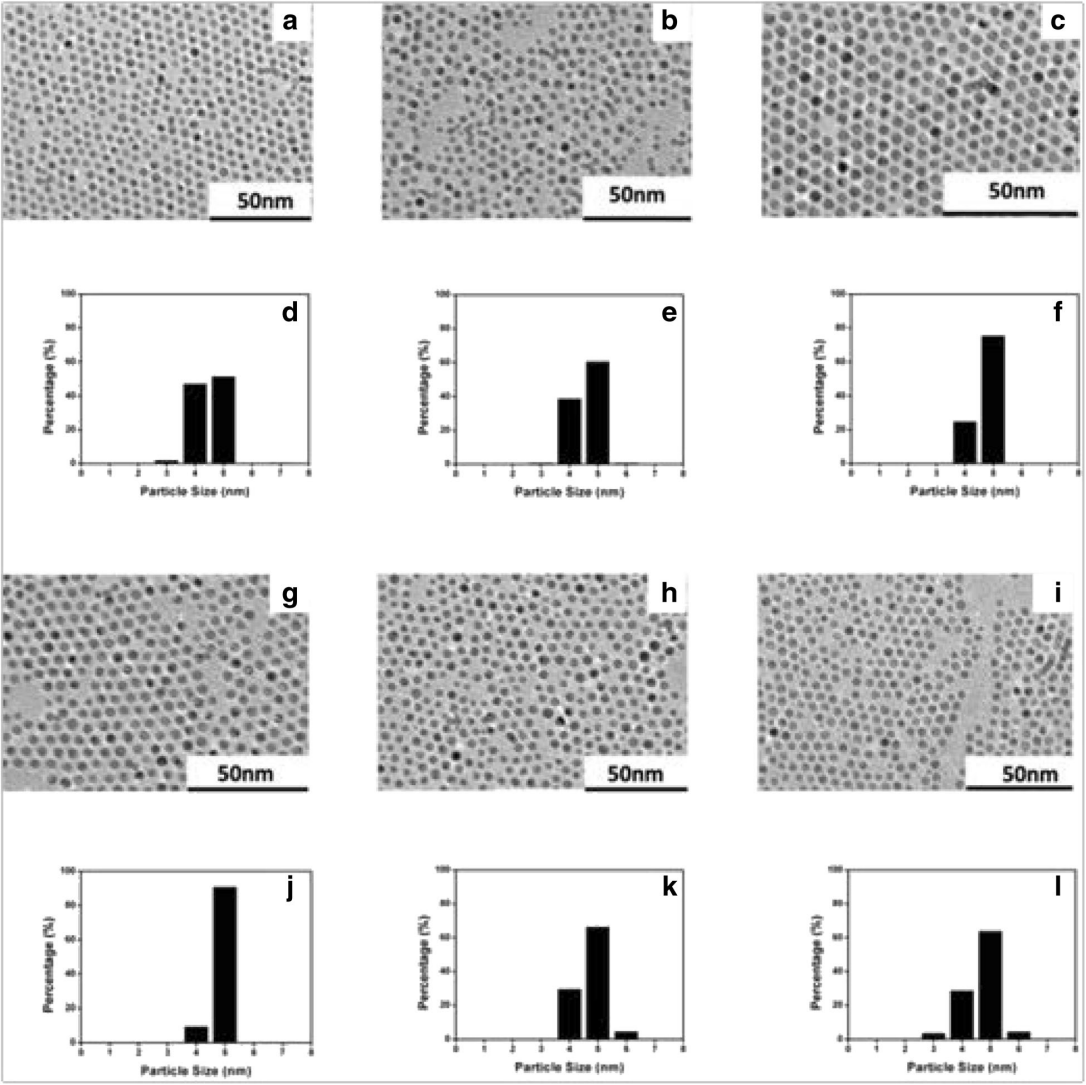
TEM images of the Au NPs after digestive ripening in chlorobenzene for different reflux times: **a** 4, **b** 8, **c** 12, **g** 16, **h** 20, and **i** 24 h. The corresponding particle size histograms: **d** 4, **e** 8, **f** 12, **j** 16, **k** 20, and **l** 24 h

**Fig. 4:**
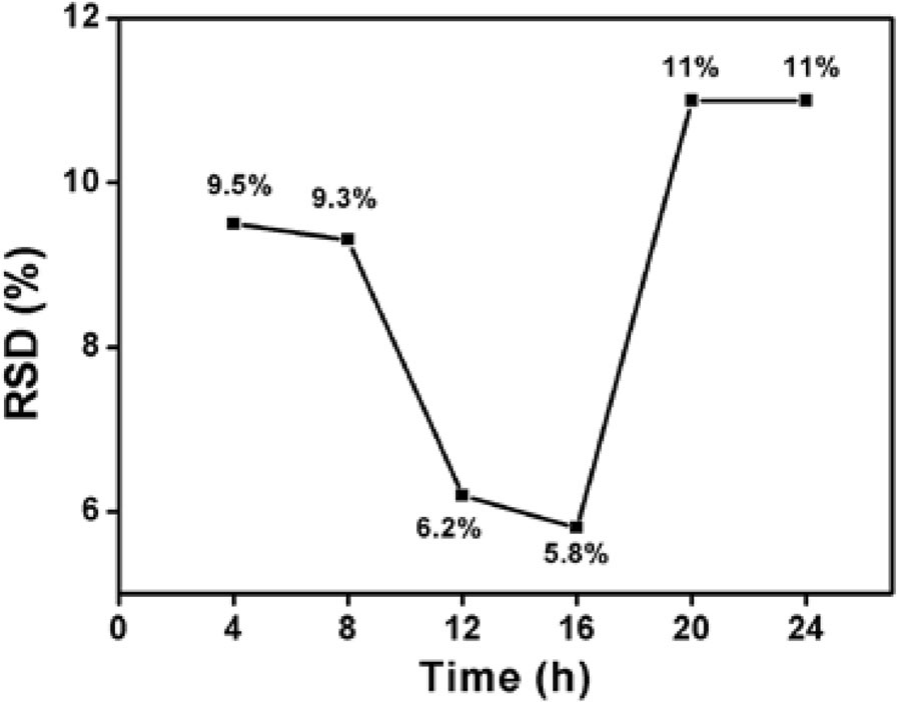
Distribution of Au nanoparticles vs. digestive ripening reflux times at 136 °C

To further improve the monodispersity of the Au NPs, the influence of the digestive ripening time was investigated. According to the above results, the optimal solvent of chlorobenzene was utilized as the reflux solvent in the following time experiment. The TEM images of the Au NPs after digestive ripening in chlorobenzene for different times were shown in Fig. 3. According to TEM observation, the corresponding size distribution was calculated, and the relationship between it and the digestive ripening time was plotted in Fig. 4. The size distribution of Au NPs had an obviously decrease in the period of the beginning 4–16 h. The results conformed to the process of digestive ripening. When polydispersed particles are heated with additional DDT, the atoms or clusters from the bigger particles are etched out by DDT, resulting in the size-reduction of particles, and these etched out atom clusters are redeposited onto the smaller particles, increasing their size. As a result, these polydispersed particles got converted to a uniform size [24]. In our experiments, those two competitive reactions achieve equilibrium in 16 h when the minimal size distribution was obtained. However, after 16 h, with the heating time, the distribution of Au NPs was increased; that could not be explained by the theory of digestive ripening any more. We speculated the possible reason was that the structure of digestive ripening agent (DDT) was damaged to some extent by the long time reflux at high temperature.

To verify the hypothesis, we designed two scenarios. One was the addition of new DDT to the system during the digestive ripening process; the other was reducing the digestive ripening temperature. In the first one, 2.5 μL new DDT was added to the solution of the Au NPs after reflux at 136 °C for 12 h, followed by another 12 h reflux. As shown in Additional file 1: Figure S1, the size distribution was only 7.0%, much lower than that of Au NPs after continuous reflux for 24 h. It suggested the new DDT took effective digestive ripening, implying the original DDT in the solution lost its effectiveness. In the other case, the heating temperature was decreased from 136 to 70 °C. As shown in Additional file 1: Figure S2 and Figure S3. The equilibrium time of digestive ripening can be achieved in 20 h, and afterwards, the size distribution of Au NPs nearly kept constant at least for another 12 h. It suggested that it was the high temperature that damaged the DDT. The two experiments confirmed the assumption and moreover demonstrated that the temperature and time made a difference to digestive ripening.

**Fig. 5:**
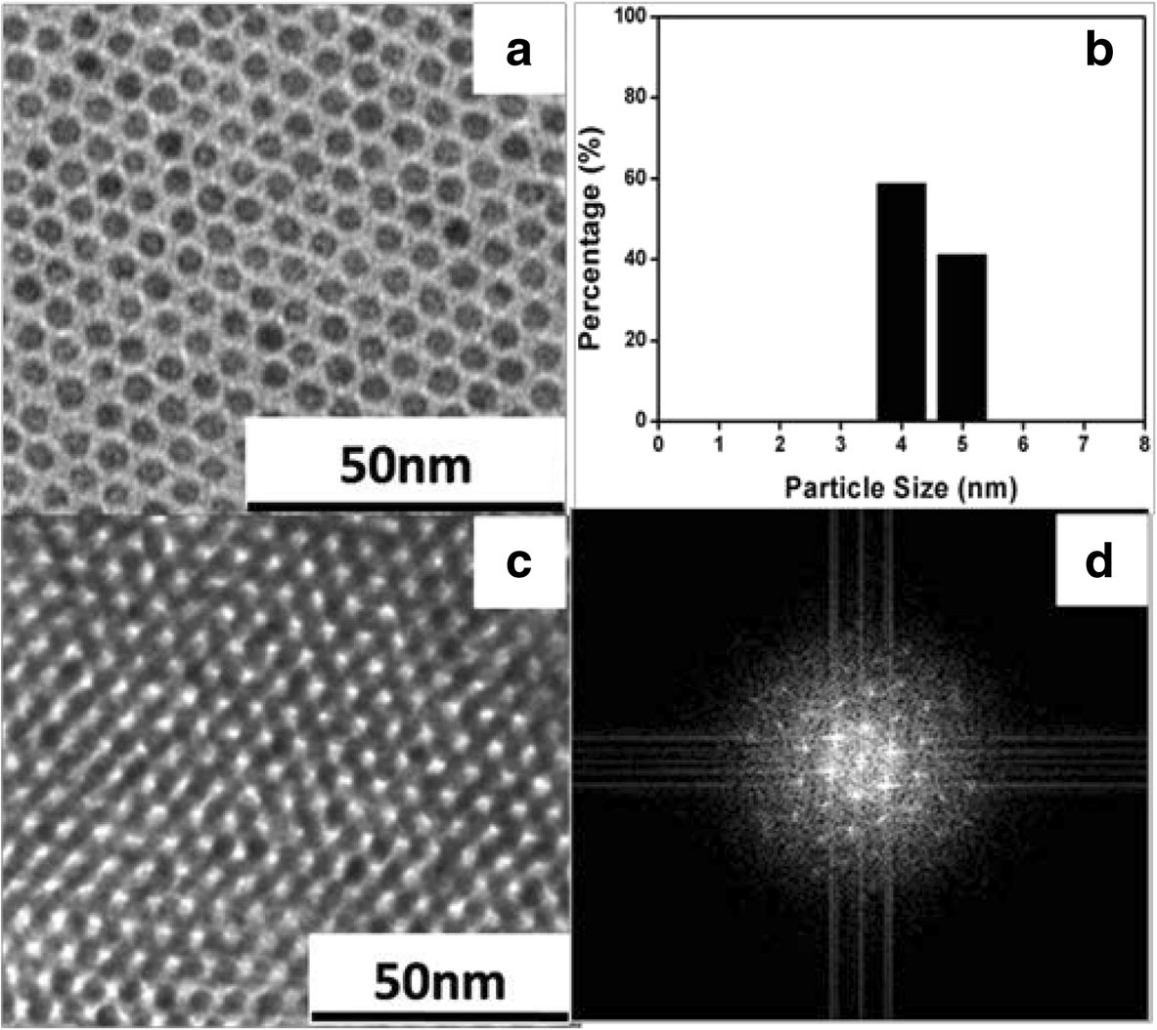
Au nanoparticles obtained in *p*-chlorotoluene solvent by reflux at 136 °C for 16 h. **a** TEM image. **b** The corresponding particle size histogram. **c** The superlattices image. **d** The corresponding fast fourier transform

The above study suggested that in digestive ripening, high solvent polarity and appropriate combination of temperature and time was crucial to the nanoparticles distribution. Based on the obtained experiments, we chose a high polarity benzol solvent, *p*-chlorotoluene (whose dielectric constant is 6.25 [25]), as the solvent to perform digestive ripening at 136 °C for 16 h. The TEM image and corresponding size distribution histogram were depicted in Fig. 5a, b. As desired, the high-quality monodisperse Au NPs was obtained, and the relative standard deviation could reach as low as 4.8%. Through simple solvent evaporation, the monodisperse nanoparticles could be self-assembled into the superlattices as shown in Fig. 5c, d. This result confirmed the importance of the solvent in digestive ripening and showed the promise of the method.

## Conclusions

This work was focused on the influence of the solvents on the size distribution of the nanoparticles in digestive ripening. The experiments indicated that for the benzol solvents, the distribution could decrease remarkably with the increase of the polarity. This could be interpreted by the Gibbs free energy of a spherically charged particle. Higher solvent polarity could lead to lower energy of the nanoparticles, which may consequently reduce the polydispersity of the nanoparticles. Through adjusting the solvent polarity, the high-quality Au NPs with 4.8% relative standard deviation and corresponding supperlattices have been achieved. Verifying the effectiveness of the method in synthesis and self-assembly. Combined with simple and facile synthesis method, the digestive ripening in an appropriate solvent could be a promising and practical approach to obtain monodispersed nanoparicles, having great potential in the nanoscience.

## Additional file

Additional file 1: Figure S1. Effect of new DDT addition into the digestive ripening. (a) TEM image. (b) The corresponding particle size histogram. The particle size distribution obtained is 4.31 ± 0.30 nm and the relative standard deviation is 7.0%. Figure S2: TEM images of the Au nanoparticles at 70 °C in different times (a) 12, (b) 16, (e) 20, (f) 28, and (g) 32 h. The corresponding particle size histograms: (c) 12, (d) 16, (h) 20, (i) 28, and (j) 32 h. The particle size distributions are 4.16 ± 0.54 nm at 12 h, 3.76 ± 0.31 nm at 16 h, 4.51 ± 0.31 nm at 20 h, 4.30 ± 0.25 nm at16 h, 4.58 ± 0.31 nm at 28 h, and 4.60 ± 0.30 nm at 32 h. Figure S3: Distribution of Au nanoparticles vs. digestive ripening reflux times at 70 °C. (DOCX 3280 kb)
