# Supplementary material for: Solvent: A Key in Digestive Ripening for Monodisperse Au Nanoparticles
Source: Nanoscale Res Lett. 2017 Jan 9;12:25. doi: 10.1186/s11671-016-1797-7 (PMC5222767; doi:10.1186/s11671-016-1797-7)
Supplement: Additional file 1: Figure S1. — Effect of new DDT addition into the digestive ripening. (a) TEM image. (b) The corresponding particle size histogram. The particle size distribution obtained is 4.31 ± 0.30 nm and the relative standard deviation is 7.0%. Figure S2: TEM images of the Au nanoparticles at 70 °C in different times (a) 12, (b) 16, (e) 20, (f) 28, and (g) 32 h. The corresponding particle size histograms: (c) 12, (d) 16, (h) 20, (i) 28, and (j) 32 h. The particle size distributions are 4.16 ± 0.54 nm at 12 h, 3.76 ± 0.31 nm at 16 h, 4.51 ± 0.31 nm at 20 h, 4.30 ± 0.25 nm at16 h, 4.58 ± 0.31 nm at 28 h, and 4.60 ± 0.30 nm at 32 h. Figure S3: Distribution of Au nanoparticles vs. digestive ripening reflux times at 70 °C. (DOCX 3280 kb) [file 11671_2016_1797_MOESM1_ESM.docx]

**Supplementary files**

Solvent: a key in digestive ripening for monodisperse Au nanoparticles

Peng Wang^a#^, Xuan Qi^a#^, Xuemin Zhang^a^, Tieqiang Wang^a^, Yunong Li^a^, Kai Zhang^a^, Shuang Zhao^a^*, Jun Zhou^a,b^* and Yu Fu^a^*

^a^ *College of Sciences, Northeastern University, Shenyang 110004, China*

^b^ *School of Materials Science and Engineering, Key Laboratory for Anisotropy and Texture of Materials, Ministry of Education, Northeastern University, Shenyang 110004, China*

^*^ Correspondence should be addressed to Shuang Zhao, Jun Zhou and Yu Fu

E-mail: [shuangzhao@aliyun.com](mailto:shuangzhao@aliyun.com), hafouniu@126.com, fuyu@mail.neu.edu.cn

^#^ These authors contributed equally to this work


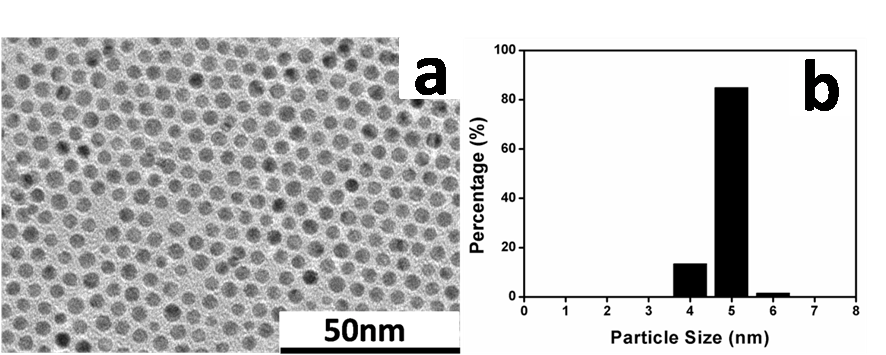


Fig. S1 Effect of new DDT addition into the digestive ripening. (a) TEM image, (b) the corresponding particle size histogram. The particle size distribution obtain is 4.31±0.30 nm and the relative standard deviation is 7.0%.


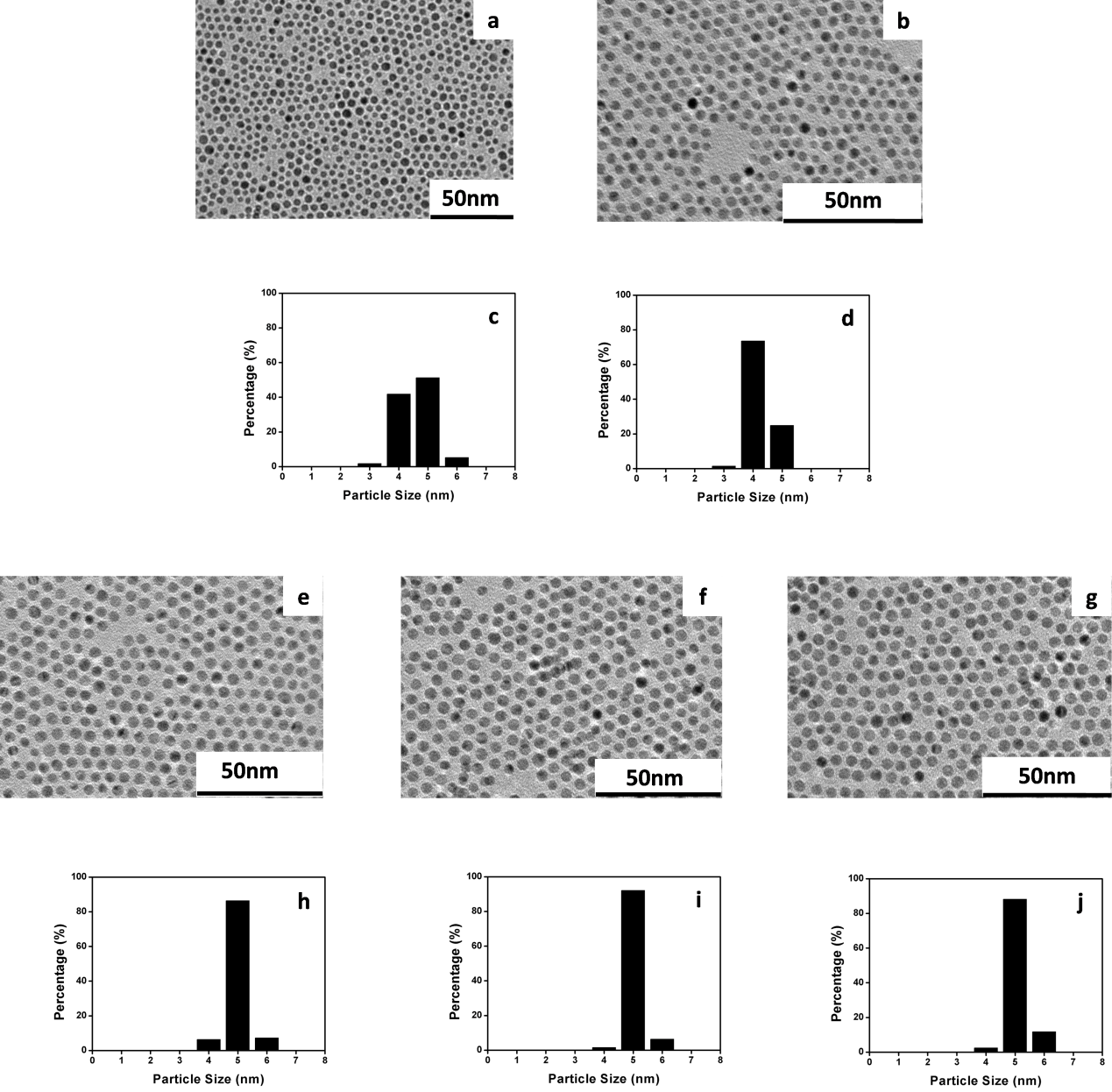
**Fig. S2** TEM images of the Au nanoparticles at 70 ^o^C in different times (a) 12, (b) 16, (e) 20, (f) 28, and (g) 32 h. The corresponding particle size histograms. (c) 12, (d) 16, (h) 20, (i) 28, and (j) 32 h. The particle size distributions are 4.16±0.54 nm at 12 h, 3.76±0.31 nm at 16 h, 4.51±0.31 nm at 20 h, 4.30±0.25 nm at16 h, 4.58±0.31 nm at 28 h and 4.60±0.30 nm at 32 h.





**Fig. S3** Distribution of Au nanoparticles v.s. digestive ripening reflux times at 70^o^C.
